# Reprogramming the tumor microenvironment: methylmalonic acid at the intersection of ccRCC metabolism and macrophage polarization

**DOI:** 10.1038/s41418-026-01685-x

**Published:** 2026-02-14

**Authors:** Jonatan Barrera-Chimal, Casimiro Gerarduzzi

**Affiliations:** 1 Centre de Recherche de l’Hôpital Maisonneuve-Rosemont, Centre affilié à l’Université de Montréal, Montréal, QC Canada; 2 Département de Médecine, Faculté de Médecine, Université de Montréal, Montréal, QC Canada

**Keywords:** Cancer metabolism, Tumour biomarkers

Clear-cell renal cell carcinoma (ccRCC) is often described as a “metabolic disease”, a malignancy that gets its name and biological identity from the transparent lipid droplet formations within its cytoplasm. Rather than being a simple phenotype, this metabolic alteration is a dominant mechanism of tumorigenesis and therapy resistance, driven by multiple mutations (e.g., in VHL, FH, SDH) that converge on deregulated oxygen- and nutrient-sensing, mitochondrial dysfunction and rewired metabolism [1, 2]. Key features include increased glycolysis (Warburg effect), up-regulation of the pentose-phosphate pathway, lipid anabolism and storage, down-regulated TCA cycle enzyme expression (e.g., ACO2, SUCLG1) and fatty acid oxidation suppression [2, 3]. As a result of the altered metabolism, specific metabolites, or so-called “oncometabolites”, accumulate and actively drive tumor progression while modulating the tumor microenvironment (TME) [4]. In the case of ccRCC, metabolic reprogramming is especially pronounced and integral to pathogenesis. This metabolic complexity was recently revealed to have another layer of regulation by Miao et al. in their current *Cell Death & Differentiation* publication [5]. In this thorough study, they elegantly reveal a previously underappreciated oncometabolite, methylmalonic acid (MMA), having a multicellular effect in the tumor microenvironment by directly influencing ccRCC cells and reprogramming macrophage polarization, which leads to the production of tumorigenic factors such as VEGFs. More impressively, they go on to show that the metabolic derailment could be corrected by simply feeding a low-branched-chain-amino-acid (BCAA) diet, which diminishes tumor progression in pre-clinical models; moreover, the efficacy of the treatment is improved with a methylmalonylation-blocking agent (MC3138).

## From metabolic debris to oncometabolite

We first came to understand the importance of MMA through inborn errors of metabolism. One such example is a loss-of-function mutation in methylmalonyl-CoA mutase (MMUT), a mitochondrial enzyme required for MMA metabolism. Consequently, substrate accumulation leads to methylmalonic acidemia, where the excess MMA exposes a novel post-translational modification known as methylmalonylation that can inactivate its target enzymes and thereby contribute to profound multi-organ manifestations [6]. In parallel fashion, Miao et al. [5] took this concept into ccRCC, showing that lower levels of MMUT are recurrently observed in ccRCC patients and cells. Accordingly, they carried out metabolic profiling on paired tumor and adjacent ccRCC tissues and found MMA to be highly elevated in tumors and markedly increased in the circulation of ccRCC patients. In a natural type of “rescue” experiment, the authors elegantly showed these MMA levels to drastically drop post-nephrectomy, implicating their mechanistic importance in tumorigenicity.

Although MMUT loss disrupts MMA metabolism, this accumulation is not merely a by-product, as the authors provide ample evidence that MMA exerts a signaling profile reminiscent of other well-described oncometabolites in ccRCC and other tumors, such as fumarate, succinate, and 2-hydroxyglutarate. Unlike these oncometabolites, MMA has only recently emerged and remains underexplored in the context of ccRCC. From the growing body of evidence on MMA, we know that its systemic levels increase with age, vitamin B12 deficiency, and tumor-driven metabolic dysregulation. In a landmark study, it was shown that higher MMA concentrations induce a pro-aggressive phenotype in cancer cells through activating the TGFβ signaling and the induction of the transcription factor SOX4, ultimately leading to an EMT-like phenotype [7]. Moreover, MMA activates tumor-associated fibroblasts, which in turn release IL-6-loaded extracellular vesicles, which then act on tumor cells via JAK/STAT3 and TGFβ pathways, promoting drug resistance and metastatic progression, highlighting its potential to modulate the TME [8]. A further study reported that MMA exposure reduced mitochondrial NADH regeneration, induced T-cell exhaustion and suppressed anti-tumor immunity in vitro and in vivo [9]. These observations support the concept from Miao et al. [5] that MMA may function as an oncometabolite in ccRCC, actively contributing to remodeling the TME through its actions on tumor cell aggressiveness features and macrophage polarization.

## Novel mechanistic findings: a methylmalonylated switch in Hedgehog signaling

**Fig. 1:**
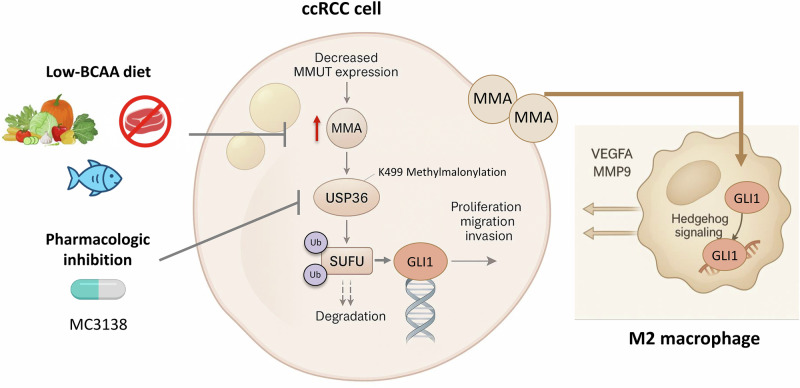
MMA-driven USP36 methylmalonylation activates Hedgehog signaling and remodels the tumor microenvironment. Reduced expression of MMUT in ccRCC cells causes accumulation of methylmalonic acid (MMA), which methylmalonylates USP36 at K499 and inhibits its deubiquitinase activity. Impaired USP36 function increases SUFU ubiquitination and decreases SUFU SUMOylation, triggering its proteasomal degradation. Reduced SUFU levels release GLI1, thereby enhancing Hedgehog pathway activation and promoting ccRCC cell proliferation, migration, and invasion. Extracellular MMA further shapes the tumor microenvironment by activating GLI/Hedgehog signaling in tumor-associated macrophages, driving their polarization to an M2 phenotype that secretes pro-tumorigenic mediators, including VEGFA and MMP9. Proposed therapeutic strategies include a low-BCAA diet to reduce the bioavailability of MMA and pharmacological inhibition of USP36 methylmalonylation using MC3138. Created with BioRender.com.

According to the study by Miao et al. [5], decreased expression of MMUT was associated with a negative prognosis in ccRCC. From a functional perspective, irrespective of the mechanism, MMA has the potential to promote tumor growth by increasing the proliferation, migration, and invasion of cancer cells in a dose-dependent manner, while the restoration of MMUT expression reverses these effects. In addition, MMA enhances tumor progression by stimulating M2 macrophages, which in turn secrete pro-tumor factors MMP9 and VEGFA. As demonstrated by RNA-seq and luciferase assays, the mechanistic centerpiece of the study is the discovery that MMA activates the Hedgehog signaling pathway, primarily via methylmalonylation of the deubiquitinase USP36 at lysine 499 (K499), thereby inhibiting its catalytic activity. Such modification on USP36 upsets its delicate balance of ubiquitination versus SUMOylation of SUFU, an inhibitor of GLI1. Consequently, SUFU is ubiquitinated and degraded, releasing GLI1 to promote Hedgehog signaling. This is evidenced by the USP36 mutation at K499, which prevents the destabilizing effect. The resulting altered tumor microenvironment is sustained on a multicellular Hedgehog/GLI axis that creates a self-amplifying circuit of proliferating signals in ccRCC cells and macrophage polarization into a pro-tumorigenic M2 phenotype (Fig. 1). Overall, the findings suggest that targeting MMA accumulation and its downstream signaling offers a promising strategy for ccRCC treatment.

The conceptual advance comes from identifying methylmalonylation as a post-translational modification (PTM) that alters the activity of deubiquitinases. Even if acylation of lysines, such as succinylation and malonylation, has been mentioned [10, 11], the methylmalonylation modification is novel because it connects amino acid metabolism and the control of methylmalonylated proteins. This metabolism-PTMs connection offers the enticing proposition of having tumor metabolites as active signaling molecules.

## Turning metabolism against the tumor

If MMA fuels malignancy, could its depletion starve it? Miao et al. [5] take their fundamental concepts and apply them in a clever intervention strategy. They took inspiration from the clinical dietary management of patients with methylmalonic acidemia and placed ccRCC-bearing mice under a low-BCAA diet to reduce MMA availability. This simple dietary change worked therapeutically to not only diminish tumor growth but also reduce pulmonary metastases and limit M2 macrophage infiltration. This reinforces the idea that a net metabolic shift in the diet can therapeutically restrain the immune system, and whether MMA could contribute to immune evasion or resistance to immunotherapies should be investigated.

Therapeutic nutrient restriction has often been considered impractical, yet recent studies on serine/glycine and methionine metabolism show that selective metabolic vulnerabilities can provide opportunities for therapeutic exploitation [12, 13]. The low-BCAA diet discussed in this context will also be a feasible clinical adjunct to treatment for many, and BCAA restriction will be less demanding for patients, as it can be transient and modulated as needed.

Although elegant, there are a number of unanswered questions. First, what is the mechanistic reason for the observed levels of MMUT being decreased in ccRCC. Second, proteomic screens have identified numerous methylmalonyl-lysine sites [14]; thus, metabolic off-targeting may have wide-ranging effects on metabolism. On the translational side, there are several important questions: (1) Is a measurement of MMA levels useful as a biomarker of aggressive disease, or as a measure of changes in the tumor microenvironment (TME) or metabolic vulnerability to therapy? (2) Where is MMA spatially distributed within the tumor and stroma? (3) How practical is a low-BCAA diet as a therapeutic approach?

On the clinical side, the use of BCAA restriction as a form of therapy in human patients with ccRCC is a long way from being practiced due to the metabolic and skeletal muscle comorbidities that are present in RCC patients. The drug MC3138 is effective in mice, but MC3138 has not been evaluated regarding its pharmacokinetic profile or its toxicity in humans. As such, the concept of “metabolic detuning”, or lowering the production of a metabolite that is toxic to the cell, is certainly an interesting approach that may be used to develop new combinations of therapies, including those using immune checkpoint inhibitors or anti-angiogenic drugs.

In summary, the current study clearly demonstrates that ccRCC is a “metabolic” disease and not simply a result of signaling pathway alterations. In addition, this work further demonstrates that the metabolic landscape of tumors is not random and may reflect the cellular wiring of the tumor cells, which may provide additional points of intervention beyond the information provided by genomic studies.
